# Coalescent Simulations and Field Experiments Support Natural Selection as the Driving Force Maintaining Color Differences Between Adjacent Populations of *Ceroglossus chilensis* (Coleoptera: Carabidae)

**DOI:** 10.3390/insects17010044

**Published:** 2025-12-30

**Authors:** Benjamín Arenas-Gutierrez, Antonio Rivera-Hutinel, Carlos P. Muñoz-Ramírez

**Affiliations:** 1 Programa de Magíster en Ciencias Mención Entomología, Instituto de Entomología, Universidad Metropolitana de Ciencias de la Educación, Av. José Pedro Alessandri 774, Ñuñoa, Santiago 7760201, Chile; benjamin.arenas2023@umce.cl; 2 Vicerrectoría de Investigación e Innovación, Universidad Arturo Prat, Avenida Arturo Prat 2120, Iquique 1110939, Chile; 3 Instituto de Entomología, Universidad Metropolitana de Ciencias de la Educación, Av. José Pedro Alessandri 774, Ñuñoa, Santiago 7760201, Chile; antonio.rivera@umce.cl

**Keywords:** aposematic beetles, Müllerian mimicry, southern South America, COI, phenotypic diversity

## Abstract

Determining the drivers of phenotypic evolution and their role on shaping regional phenotypic diversity is a fundamental aspect of mimicry research. Beetles of the genus *Ceroglossus* are well known for their rampant color diversity, and species have been shown to exhibit convergence in elytral coloration when in sympatry. Despite solid phylogenetic evidence showing that color convergence is not the result of shared ancestry, more direct evidence of the role of selection on color evolution is lacking. In this study, we used two different, but complementary approaches to test whether selection plays a role in color divergence and whether predators are responsible for its maintenance. We first used molecular data to assess gene flow. Subsequently, we used the sequence data to conduct coalescent simulations and generate expectations for color fixation under the hypothesis of genetic drift. Second, we conducted field experiments to test whether predators exhibited different preferences on mimetic versus non-mimetic phenotypes. Results from the coalescent simulations showed that color fixation exhibited strong deviations from the genetic drift expectation, implying that selection must be responsible for color divergence. Field experiments showed that translocated beetles (into areas where they were not mimics) suffered higher rates of attacks than those beetles that mimicked the locally abundant phenotypes. Thus, our study supports the hypothesis of selection as a driving force of color evolution in *Ceroglossus* beetles and provides the context that explains and supports previous claims of Müllerian mimicry.

## Simple Summary

Observable characteristics of organisms can evolve by different mechanisms such as genetic drift and selection. In this study, we aimed to assess which of these forces are responsible for the differences in color between various adjacent populations of a ground beetle species, *Ceroglossus chilensis*, using genetics and field experiments. Our approach supported selection as the main force driving color differences and showed that predators are the main selective force. This study contributes to the understanding of the processes driving diversity in *Ceroglossus chilensis* and provides insights regarding which processes may be involved in the pattern of mimicry observed within the genus.

## 1. Introduction

Elucidating the processes that drive and maintain phenotypic diversity is one of the main goals of ecology and evolutionary biology [1,2]. Within a species, the main forces driving the evolution of phenotypic variation are mutations, gene flow, natural selection, and genetic drift [3]. Although these forces are the subject of comprehensive research, disentangling the processes responsible for striking phenotypic variation in natural populations remains challenging.

**Figure 1:**
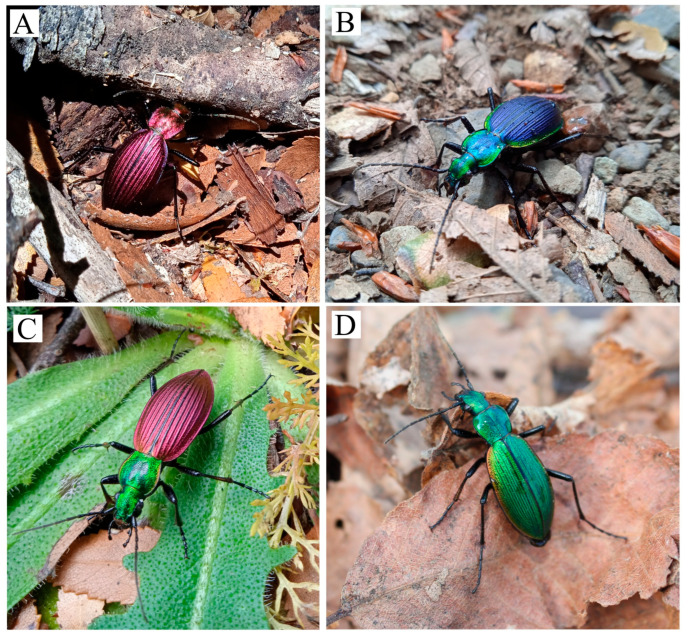
Four phenotypes of *Ceroglossus chilensis* from Curacautín, South Chile (Araucanía Region) in their natural habitat. (**A**), Red phenotype. (**B**), Blue phenotype. (**C**), Red-Green phenotype. (**D**), Green phenotype.

Ground beetles in the genus *Ceroglossus* exhibit exceptional color variation across temperate forests of southern South America [4], which make them suitable models to study on questions about phenotypic evolution [5,6]. The low number of species described (only ten species) remarkably contrasts with more than 90 subspecies described [4], which account for the high and geographically structured color diversity found at the intraspecific level. For example, within *Ceroglossus chilensis*, approximately 28 subspecies have been described along its more than 1200 km latitudinal range (35S to 47S). At the population level, color is typically monochromatic within a given population but varies greatly across populations from different geographic areas [4] (Figure 1). Color in these beetles is not pigment-based, but structurally-based, originating from the physical microstructure of the external layer of the cuticle [7]. Thus, it can be highly heritable and prone to selection as shown for other insects [8,9].

One puzzling aspect of these beetles’ geographic color variation is that no apparent barriers separate the distinct color phenotypes. In the absence of barriers to gene flow, theory predicts that gene flow should counteract phenotypic divergence [10]. How are these phenotypes maintained in the presence of potential gene flow? One possible explanation is that color plays a role in local adaptation, so strong natural selection can maintain color differences between close geographic areas, even in the presence of gene flow [11].

Like most members of the family Carabidae, *Ceroglossus* species are chemically defended [4,12] (synthesis occurs in pygidial glands), and when disturbed, they discharge deterrent substances including acetic, propanoic, tiglic, and methacrylic acids [12]. The bright, metallic coloration of most *Ceroglossus* species contrasts starkly with the background substrate, suggesting that their coloration is for warning (i.e., aposematic color) rather than crypsis [13]. In spite of this high geographic variation in color, *Ceroglossus* species that co-occur within a geographic area often share the same coloration [13,14]. That is, on average, species tend to be phenotypically more similar to other *Ceroglossus* living in the same locality than they are to conspecific populations encountered in more distant locales. Furthermore, phylogenetic work has shown that color-matching in *Ceroglossus* is not the product of shared ancestry [13,14,15]. Instead, similar color morphs have evolved repeatedly and independently across the phylogeny, a pattern that is commonly considered as strong support for convergence in general [16], and for mimicry in particular [17,18].

Müllerian mimicry theory predicts strong selection by predators for a single aposematic signal due to positive frequency-dependent selection [19,20]. That is, the fitness of a phenotype is higher when it is abundant and lower when it is rare. Predators learn to avoid the locally common and abundant aposematic phenotype by sampling prey until a threshold number is reached, after which predators develop an aversion response. This learning process is aided by the recognition of a typically conspicuous warning signal, such as bright coloration, which can then be remembered and distinguished from other phenotypes—an aposematic signal [21,22]. As such, any new variant that appears in the population and departs from the locally common phenotype (e.g., a new mutation or immigrants from nearby areas) should be rapidly eliminated by predation because predators do not recognize the new phenotype as dangerous or defended [23]. Therefore, this process not only predicts locally monomorphic species, but also the maintenance of regional diversity, given that migration between areas with different aposematic phenotypes would be reduced by local predation on the incoming migrants.

In *Ceroglossus* beetles, the idea that Müllerian mimicry can maintain geographically-based color structure between populations is sound [13]. However, and beyond color matching analyses and phylogenetic testing, the hypothesis that selection underlies color diversity has not been further investigated. In this study we used coalescent simulation analyses and field experiments to complementarily test whether natural selection plays a role in maintaining geographically structured color diversity.

## 2. Materials and Methods

To test our hypothesis, we used two complementary approaches. We first tested whether genetic drift could be a possible explanation for the maintenance of the color differences between two adjacent localities (our null model). Deviations from the genetic drift expectation should support selection. This method was first proposed by [24] to test sexual selection in jumping spiders, and later used by [25] to test selection in mimetic poison frogs. We collected molecular data and conducted coalescent simulations to test whether phenotypic differentiation differed significantly from the expectation of genetic drift. Second, to assess whether predators play a relevant role in maintaining the color differences, we conducted a translocation experiment to assess whether predators exhibit preferences for beetles with different phenotypes in different areas.

### 2.1. Coalescent Simulations to Evaluate Genetic Drift

#### 2.1.1. Sampling and Lab Work to Obtain DNA Sequences

**Figure 2:**
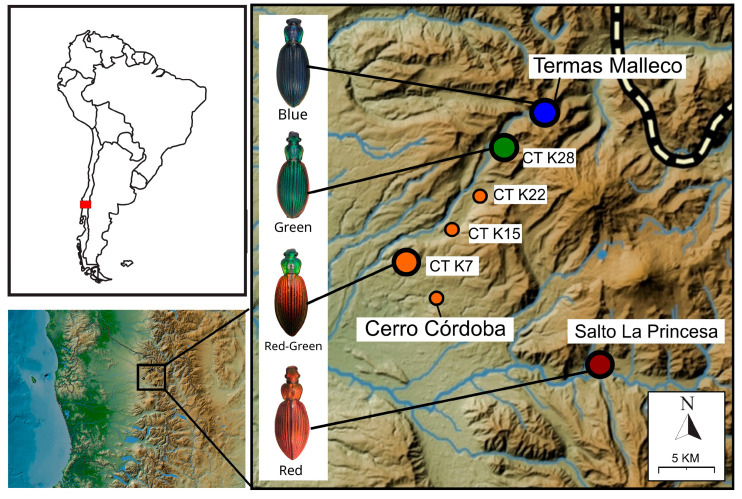
Map depicting the four phenotypes of *Ceroglossus chilensis* analyzed in this study and their geographical location in South Chile (Araucanía Region).

A total of 41 individuals of *Ceroglossus chilensis* sampled between years 2022 and 2024 and from four different sites (9–11 individuals per site) were selected for the molecular analyses. These sites are all located in an Andean region from South Chile (Curacautín, Araucania Region) which are geographically close to each other, but contain populations of *C. chilensis* that have different body coloration (Figure 1 and Figure 2 and Table 1).

DNA from the 41 individuals was extracted from legs using the QIAGEN DNeasy Blood & Tissue Kit (QIAGEN Inc., Chatsworth, GA, USA) following the manufacturer’s protocol. A portion of 658 bp of the mitochondrial gene encoding cytochrome oxidase subunit I (COI) was amplified using universal primers LCO1490 and HCO2198 [26]. Each PCR reaction contained 1 μL of extracted DNA, 2 μL of 5× buffer, 1.5 μL of MgCl_2_, 1 μL of 10 mM dNTPs, 0.4 μL of 1% BSA, 0.8 μL of each primer (10 μM), 0.06 μL of Taq DNA polymerase (Invitrogen, Waltham, MA, USA), and ddH_2_O to make a total of 25 μL reaction. A standard PCR profile with one-minute duration for each step, a total of 35 cycles, and a final extension of 10 min at 72 °C was followed. The annealing temperature was 52 °C. After checking PCR products in 1.5% agarose gels with SYBR Safe as the stain dye, successful products were purified and sequenced in Macrogen Inc. (Santiago, Chile).

#### 2.1.2. Sequence Editing and Phylogenetic Analyses

Chromatograms were edited in ChromasPro v2.2.0 (Technelysium Pty Ltd., Brisbane, Australia), aligned using the MUSCLE algorithm implemented in MEGA12 [27] and checked via amino acid coding to test for unexpected frame shift errors or stop codons. Sequences were made available in GenBank with accession numbers PX051377-PX051417.

Maximum likelihood genealogy was estimated using RAxML-NG v. 1.1.0 [28]. Best-fit models of molecular evolution were chosen by Modeltest-NG [29], using the Bayesian information criterion (BIC). The RAxML analysis was run with 1000 nonparametric bootstrap replicates, followed by a search for the best-scoring ML tree. In addition, to better visualize any putative genetic structure, a haplotype network was constructed with PopArt [30], using the minimum spanning network option. Complementary to this analysis, genetic structure between sites was also assessed by calculating the statistic *Fst* in the program Arlequin ver. 3.5 [31].

#### 2.1.3. Coalescent Simulation Procedure

First, we used Tajima’s D statistic to check for neutrality in the COI sequences (which serves as our baseline for comparison to evaluate the rate of change in our hypothetical nuclear sequences) [32]. This test compares the average level of pairwise sequence divergence for sets of sequences within populations with the number of segregating sites. Negative values indicate the action of purifying selection, a selective sweep, population expansion or a complete bottleneck, whereas a positive value is consistent with the action of diversifying selection, population mixing, or a partial bottleneck [33]. If this analysis uncovered elements of purifying selection (e.g., a negative Tajima’s D), this would make our test for selection on nuclear genes using simulations of the coalescent more conservative, as it would drive more rapid coalescence in the mtDNA haplotypes within populations. In such a scenario, selection on phenotype (pattern) must be even stronger, producing greater lineage sorting than that exhibited by the mtDNA, to detect a significant difference.

The coalescent simulation test for selection was implemented in MESQUITE version 2.6 [34] and consisted of the following steps [24,25]: (1) We estimated the phylogenetic relationships for the haplotypes from the four color morphs, and then (2) used those trees to calculate the number of steps required to constrain the gene haplotypes (haploid genotypes) within each morph (this measure is called the *s* statistic [35]). (3) Results were compared to simulated gene trees (of the same population size, phylogenetic structure, and sample size), which provides an estimate of time since divergence. Using those results, (4) we simulated the coalescence of a hypothetical nuclear locus that controls phenotype under neutral divergence and compared these results to our observed data.

Basically, we asked whether alleles at a hypothetical gene controlling color pattern have diverged more rapidly than a hypothetical nuclear gene evolving neutrally. If the estimated coalescence of a neutral gene exhibits shallow (rapid) convergence and complete lineage sorting in a high proportion of the simulations, then drift cannot be ruled out as a possible source of this divergence. However, if our neutral gene exhibits coalescence occurring on a longer timescale than the observed sorting of the phenotypic trait (body coloration, in this case), this would support the hypothesis that selection rather than drift is responsible for the level of the phenotypic divergence observed.

Following the Masta and Maddison (2002) approach [24], two different tree topologies and two different types of comparisons for the analyses of the s statistic were implemented. The topology derived from our maximum likelihood phylogenetic analysis, and a “star” topology (i.e., an unresolved polytomy) were used. This accounts for uncertainty in the phylogenetic reconstruction. Population comparisons were carried out bycomparing the four color morphs with each other simultaneously (assuming that each morph represents fixation of a distinct allele).

The values of *s* obtained from the phylogenetic analyses (step two) were compared against the values of *s* obtained from simulating gene trees (for the same sample size of gene copies), constrained in the same type of morph tree, under a specific set of conditions (i.e., a specific combination of effective population size and branch length). Effective population size was held constant (at Ne = 10,000) across all simulations. This number may not be completely accurate, but it is held constant between all analyses and hence does not bias the results. The branch lengths were fine-tuned until a conservative estimate for the maximum possible branch lengths in the actual tree was reached (i.e., branch length that produced a minimum of 5% of *s* values as low as the empirical value). 

This provides an estimate of the maximum length that branches in the simulated trees can have and still produce a value of *s* consistent with that reconstructed from the empirical gene tree. Making the branches longer represents a conservative approach as it increases the likelihood of coalescence of neutral genes within a particular branch (resulting in genotypic sorting that matches phenotypic sorting).

Soon after determination of the maximum value for the branch length, the coalescence of a hypothetical nuclear locus controlling phenotype was simulated. Because the original simulations were conducted using mtDNA genotypes (haploid DNA), the branch lengths had to be divided by four. Simulations were replicated 10,000 times. The simulated trees were then used to calculate the distribution of the *s* statistic among the simulations. An *s* value of 3 for a four-way comparison represents complete sorting among all populations. The significance of the test is dictated by the frequency distribution of *s* in the simulated dataset. For example, a value of 3 occurring at a frequency of 5% or more (for the four-way comparison) would mean we were unable to reject the null hypothesis that the divergence of the hypothetical nuclear locus is due to neutral divergence under random genetic drift. In contrast, if the value of 3 occurs less than 5% of the time, the null hypothesis that drift and not selection explains the divergence in color pattern among morphs in *C. chilensis* can be rejected.

### 2.2. Predation Experiments to Test Predator Preferences for Different Phenotypes

**Figure 3:**
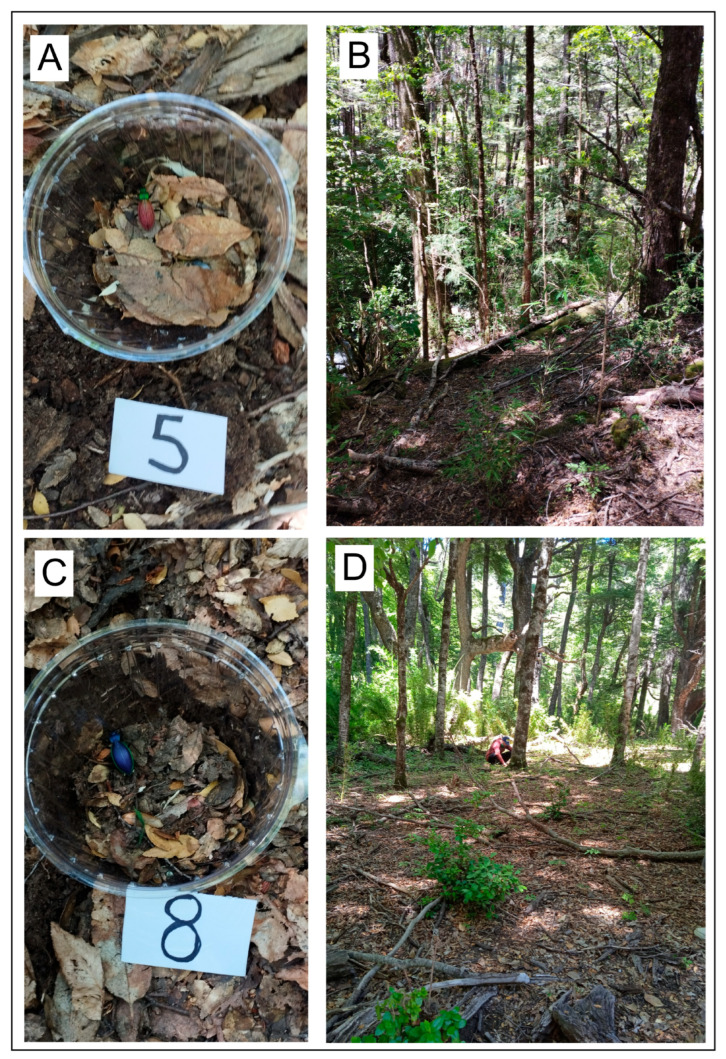
Sites used for field experiments with examples of two containers placed at each site, (**A**,**C**). (**B**), site at Cerro Córdoba. (**D**), site at Termas Malleco. A total of 100 containers were placed at each site for the translocation experiment. Numbers next to the container were used to keep track of each of the 100 containers at each site.

Given that color differences between areas may be driven by local adaptation (i.e., adaptation to different aposematic colors), we tested whether there were differences in predator attacks on different phenotypes of *Ceroglossus chilensis*, translocating beetles between areas with different aposematic colors (Figure 3). For this experiment we selected two sites harboring beetles with contrasting phenotypes. One of these sites, Cerro Cordoba, is characterized by *Ceroglossus chilensis* with red elytra and green head and pronotum (Red-Green phenotype). In the other site, Termas Malleco, the beetles are blue (Blue Phenotype) (Figure 1, Figure 2 and Figure 3). Before the experiment, several pitfall traps were placed in both areas (but in slightly different locations to avoid potential perturbations at the experiment sites), with red vinegar as bait to collect the living beetles needed for the experiment. One week later, at each of these sites, a total of 100 plastic containers were placed, 50 of them containing a single beetle with the mimetic color of that site, and the other 50 containing a beetle with the different color (translocated beetles). Over several days (4 days) these containers were checked to count how many beetles were attacked of each color. A valid attack is inferred when an individual is missing from the plastic container, or it is damaged. Data were analyzed via generalized linear models, with link logit and the binomial family, using the variable attacks (0 = no attack,1 = attacked) and the site and color (mimetic, non-mimetic) as factors. Analyses were conducted in R version 4.4.0 [36] using effect displays [37].

## 3. Results

### 3.1. Genetic and Coalescent Simulation Analyses

**Figure 4:**
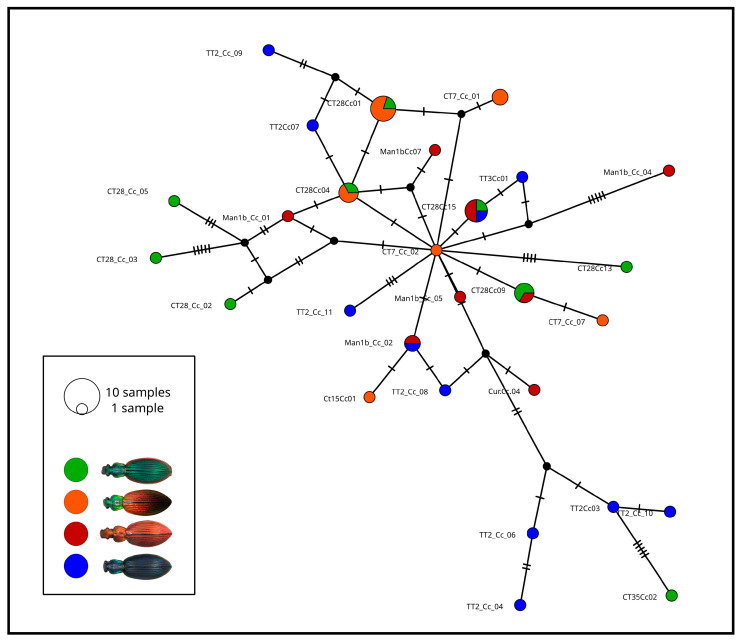
Haplotype network using 41 sequences of the mtDNA COI gene representing four color phenotypes of *Ceroglossus chilensis*. The hatch marks along the lines connecting haplotypes indicate nucleotide differences. Black circles indicate missing intermediate haplotypes.

Results from the phylogenetic analyses and the haplotype network both show a lack of genetic structure in relation to geography or color differences. The haplotype network exhibits several haplotypes with relatively similar frequencies, connected with other haplotypes by a range of 1 to 5 mutational steps; no haplogroups were evident (Figure 4). The five most frequent haplotypes (N = 2–5 individuals) were present at two or three different color morphs. Although several singleton haplotypes were unique to some sites or color morphs, these were not arranged in any geographically meaningful pattern.

Genetic differentiation as measured by the *Fst* statistic was consistent with the lack of genetic structure observed in the haplotype network and showed low values of genetic differentiation between sites, although some were significant (Table 2). Tajima’s D statistic was negative and non-significant for all phenotypes (Table 3). Tajima’s D statistic was negative and significant when pooling all sequences as one single population.

**Figure 5:**
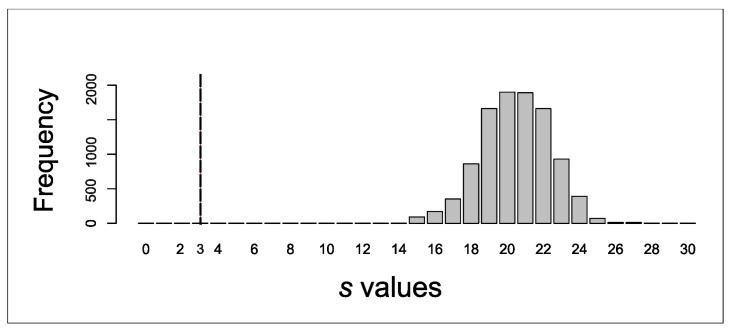
Histogram depicting the frequency distribution of simulated *s* values and the *s* value representing the case of complete sorting of the color phenotypes (vertical dashed line).

Regarding coalescent simulations, results showed that the observed s value was 19. As the two population models (bifurcating and politomy) produced similar results, only the results from the polytomy model for the population tree are described. The branch length that yielded a value of 19 for s in 5% of the coalescence simulations was 0.1105N generations, where Ne was set to 10,000 (this value was used for all populations, and hence does not influence population differences in branch length). Dividing this branch length by four (in order to simulate the coalescence of a nuclear gene controlling color pattern) yielded a branch length of 276 generations. Running the simulation with this value for the branch lengths produced an s of 14 for the lowest value, which occurred in less than 5% of the simulations. Thus, the value *s* = 3, representing complete lineage sorting (or complete reciprocal monophyly) was never reached (Figure 5). Therefore, these simulation results indicate that the observed phenotypic sorting of color morphs is extremely unlikely (*p* < 0.0001) under a model of neutral drift.

### 3.2. Field Experiments

**Figure 6:**
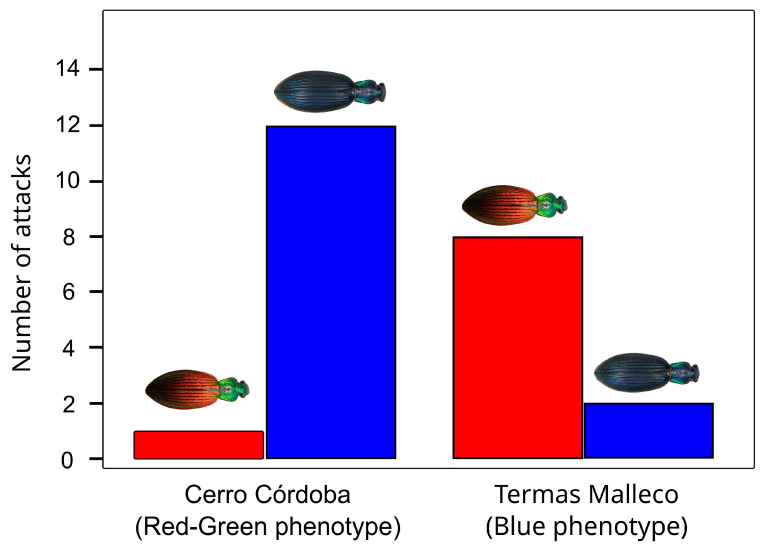
Barplot indicating the number of attacks suffered by each phenotype at two localities, Cerro Córdoba and Termas Malleco, where the local phenotype is indicated for each site in the y axis.

The experiment of reciprocal translocation showed that translocated beetles (those with phenotypes that are different from the common phenotype in the local area) were found to be attacked with higher frequency than local beetles (Figure 6). Although the number of attacks was relatively similar between the sites (chi 2 = 0.4460, df = 1, *p* = 0.5042) and between colors (chi 2 = 1.2397, df = 1, *p* = 0.2655), with a mean attack probability of 0.12 in both cases, in the Cerro Cordova site, where native beetles exhibit the Red-Green phenotype, the most attacked beetles were those that carried the Blue phenotype with a total of 12 attacks, while only one attack was recorded on beetles carrying the Red-Green phenotype. In the Termas Malleco site, where native beetles carry the Blue phenotype, the opposite trend was observed, with eight attacks to the Red-Green phenotype, and only two attacks to the Blue phenotype. This observed pattern was statistically significant indicating that the probability of attack on a specific color clearly depended on the site where it was placed (chi 2 = 15.381, df = 1, *p* = 8.79 × 10^−5^).

## 4. Discussion

The results of the two complementary approaches indicated that evolution and maintenance of color differences between geographically close populations of *Ceroglossus chilensis* is likely the product of strong selection. This result is consistent with previous studies that pointed out the remarkable convergence of elytral coloration between species [14] as well as the claim that this convergence is compelling evidence of Müllerian mimicry [13].

Phenotypic differences between geographically close areas could be the result of restricted gene flow between those areas due to, for example, a physical barrier, allowing phenotypic differentiation through genetic drift, or strong local adaptation [24]. In our study we ruled out the possibility that genetic drift alone could be the responsible force driving the phenotypic differences between the beetles. On the one hand, low genetic differentiation between color morphs as well as weak genetic structure strongly indicate pervasive levels of gene flow between areas. High levels of gene flow would rapidly homogenize any phenotypic difference, counteracting the effects of genetic drift. On the other hand, coalescent simulations showed that under none of the 10,000 scenarios simulated, the sorting of genes controlling the phenotypic differences could be complete (*s* > 3). Therefore, genetic data and coalescent theory reject the possibility that genetic drift alone could be responsible for the differences observed in *Ceroglossus chilensis*.

Although genetic analyses and coalescent simulations make clear that drift alone cannot be responsible for generating the observed phenotypic patterns, the ultimate selective force driving the patterns remain speculative at this point. Given the evidence pointing to Müllerian mimicry as a potential explanation for color convergence between species [13] and that coloration of the beetles seems aposematic (i.e., conspicuous coloration coupled with chemical defenses [12]), a reasonable hypothesis is that selection driven by predators is the responsible force driving phenotypic differences [22,23]. Our results from the field experiments supported this hypothesis. They show that translocated phenotypes, those that were not common in the area, were attacked more than the phenotypes that were common in the area. This suggest that local predators have developed a natural aversion for a particular phenotype (the locals), but were not familiar with the translocated individuals, so they attacked them more. Hence, this differential rate of attacks for different phenotypes supports the aposematic role of color and provides evidence for Müllerian mimicry in this beetle system.

Our results are consistent with similar studies in other Müllerian mimicry radiations in that predators were found to play a central role in the evolution of aposematic traits. For example, experimental studies using artificial butterflies found lower attack rates on butterflies that matched local co-mimics [38,39]. Similarly, using plasticine models to test selection for mimicry in Peruvian poison frogs [40], the authors found that local avian predators discriminated between local versus novel morphs and that local morphs experienced a significantly lower rate of predation.

One question that remains unanswered in our study is the identity of the predators. Previous studies recorded *Ceroglossus* beetles as prey items for several vertebrates, including birds [41,42,43] and mammals [44]. However, which specific predator or predators are driving the selection for mimicry in *Ceroglossus* is still unknown. This is a challenging question that has remained elusive even in the best-known mimicry systems [22]. Future studies should attempt to identify which predators are driving mimicry in *Ceroglossus* to attain a better understanding of this study system.

This study provides important information regarding the forces contributing to regional diversity in *Ceroglossus* beetles. It shows that strong selection plays an important role generating striking patterns of color structure, despite high gene flow between geographically close areas. Along with previous studies supporting color convergence in this beetle system [13,14], this study provides evidence that natural selection, via predation, is a relevant force underlying color divergence and mimicry.

## Figures and Tables

**Table 1:** Sampling and fieldwork sites with vouchers of specimens used for phylogenetic analyses.

Localities	Latitude	Longitude	Phenotype	Vouchers
Termas Malleco	−38.2325	−71.7283	Blue	TT2Cc01-04, 6–11; TT3Cc01
Camino Tolhuaca Km. 28	−38.2611	−71.7577	Green	CT28Cc01-05; CT28Cc09,13,15; CT35Cc01-02
Camino Tolhuaca Km. 22	−38.3072	−71.7847	Red-Green	CT22Cc07
Camino Tolhuaca Km. 15	−38.3478	−71.8344	Red-Green	CT15Cc01-04
Camino Tolhuaca Km. 7	−38.3730	−71.8984	Red-Green	CT7Cc01-02, CT7Cc04-07
Cerro Córdoba	−38.4033	−71.8360	Red-Green	NA
Salto de la Princesa	−38.4747	−71.6758	Red	Man1bCc01-05, Man1bCc07-08, Man1bCc10, CurCc04

**Table 2:** Genetic differentiation based on *Fst* calculations between phenotypes. * Statistically significant values based on 1000 permutations.

Phenotypes	Red-Green	Green	Red-Red
Red-Green			
Green	0.057 *		
Red	0.124 *	0.012	
Blue	0.160 *	0.056 *	0.033

**Table 3:** Basic population-level summary statistics. N = sample size, S = number of polymorphic sites, π = average number of nucleotide differences, D = Tajima’s statistic. *p*-values were calculated for the Tajima’s D statistic based on 1000 permutations.

Phenotypes	N	S	π	D	*p*-Value
Red-Green	11	7	2.14	−0.415	0.361
Green	10	25	6.62	−1.193	0.114
Red	9	15	3.69	−1.595	0.051
Blue	11	20	5.64	−0.791	0.225

## Data Availability

Sequences are available in GenBank with accession numbers PX051377-PX051417. The original contributions presented in this study are included in the article. Further inquiries can be directed to the corresponding author.
